# The effectiveness and cost-effectiveness of shared care: protocol for a realist review

**DOI:** 10.1186/2046-4053-2-12

**Published:** 2013-02-12

**Authors:** Rebecca Hardwick, Mark Pearson, Richard Byng, Rob Anderson

**Affiliations:** 1 PenTAG, Institute of Health Services Research, University of Exeter Medical School, University of Exeter, Veysey Building, Salmon Pool Lane, Exeter EX2 4SG, UK; 2 Primary Care Group, National Institute for Health Research Collaborative for Leadership in Applied Health Research and Care for the South West Peninsula, Peninsula Medical and Dental School, Plymouth University, Plymouth, UK

**Keywords:** Shared care, Long-term conditions, Cost-effectiveness, Economic evaluation, Realist review, Integrated care, Primary care

## Abstract

**Background:**

Shared care (an enhanced information exchange over and above routine outpatient letters) is commonly used to improve care coordination and communication between a specialist and primary care services for people with long-term conditions. Evidence of the effectiveness and cost-effectiveness of shared care is mixed. Informed decision-making for targeting shared care requires a greater understanding of how it works, for whom it works, in what contexts and why. This protocol outlines how realist review methods can be used to synthesise evidence on shared care for long-term conditions.

A further aim of the review is to explore economic evaluations of shared care. Economic evaluations are difficult to synthesise due to problems in accounting for contextual differences that impact on resource use and opportunity costs. Realist review methods have been suggested as a way to overcome some of these issues, so this review will also assess whether realist review methods are amenable to synthesising economic evidence.

**Methods/Design:**

Database and web searching will be carried out in order to find relevant evidence to develop and test programme theories about how shared care works. The review will have two phases. Phase 1 will concentrate on the contextual conditions and mechanisms that influence how shared care works, in order to develop programme theories, which partially explain how it works. Phase 2 will focus on testing these programme theories. A Project Reference Group made up of health service professionals and people with actual experience of long-term conditions will be used to ground the study in real-life experience. Review findings will be disseminated through local and sub-national networks for integrated care and long-term conditions.

**Discussion:**

This realist review will explore why and for whom shared care works, in order to support decision-makers working to improve the effectiveness of care for people outside hospital. The development of realist review methods to take into account cost and cost-effectiveness evidence is particularly innovative and challenging, and if successful will offer a new approach to synthesising economic evidence. This systematic review protocol is registered on the PROSPERO database (registration number: CRD42012002842).

## Background

Over 15 million people are estimated to have a long-term condition in the UK [1]. Despite only making up a third of the population, people with long-term conditions account for between 50% and 80% of GP appointments and 64% of outpatient appointments [1]. The estimated costs of providing care, as it is currently delivered to people with long-term conditions, is approximately £70 million per year , making up 70% of the total budget for health care. It is predicted that the number of people with more than one long-term condition will rise by a third in the current decade [1], and so the need to find cost-effective ways of managing long-term conditions is a priority for the National Health Service (NHS). The Quality, Innovation, Productivity and Prevention (QIPP) challenge sees long-term conditions as a prime area in which productivity and innovation will improve patient care and ensure that health service resources are used in the most appropriate way [1].

A key issue that prevents better management of people with long-term conditions is the lack of communication and care coordination between different professionals and services involved in an individual’s care. Health services are often fragmented, which can lead to poor communication between professionals and the services with which they work [2]. This can result in poor quality care and poor cost outcomes in a range of ways, such as medicine management (unsafe or costly) or inefficient use of NHS staff time. Finding ways to improve the communication interfaces between services and between professionals is an important part of developing better care management of people with long-term conditions.

One of the ways to deliver better quality and more cost-effective services is to use models of care that purposefully seek to bridge the gaps between services. Since at least the 1970s shared care has been implemented as a way of achieving this outcome [3]. It was developed as a process or model to manage referral issues, poor communication and care fragmentation, as well as to release capacity in hospital outpatient clinics. It is commonly defined as the planned delivery of care with an enhanced information exchange over and above routine clinic letters [3].

Shared care often entails a shift away from hospital care and hospital-based specialists, so it may not necessarily be a way of improving the quality of care, but as a way of reducing the cost of care without any loss in care quality or safety. Therefore, since some of the motivation or rationale for introducing shared care relates to expected improvements in cost-effectiveness, any review of shared care should try to accommodate such economic aspects.

Models of shared care can be viewed as classic complex interventions, being multi-component, highly dependent on the behaviours and choices of those delivering and receiving the care, and having highly context-dependent effectiveness. This creates challenges for conducting rigorous evaluations and also for synthesising research evidence about shared care, and for which we think more explanatory and theory-driven methods (such as realist reviews) offer advantages over conventional review methods.

Partly as a result of this complexity, the current evidence base for shared care, as reviewed by conventional methods for the systematic review of effectiveness, is highly mixed and inconclusive [4]. Rather than seeking to assess whether shared care is effective or cost-effective, it is better to use review methods that seek to explain how and why shared care and related models of care delivery are more or less effective in different circumstances or for different patient groups.

### Shared care as a complex intervention

Complex interventions have been defined as interventions that are multi-component, and where outcomes are highly context dependent and also contingent on the behaviours of participants and intervention providers [5]. At another level, complex interventions themselves can often also be viewed as complex systems, intervening in the complex system of factors that cause and sustain the problems to be tackled or managed [6,7].

Models of shared care are a good example of a complex intervention, both in terms of being inherently multi-component, and because the outcomes and resource implications are likely to be modified in some way by the context (patient group, location, organisational setting and so on) and the specific way in which schemes are implemented. If they include a component of personal encouragement, training and resources (for example, manuals) to encourage greater self-care and monitoring, or if there is a designated care coordinator (for example, a practice nurse), then they also have important behavioural elements and associated cost implications.

### The current mixed evidence about shared care

The most recent Cochrane systematic review of the evidence base for shared care concluded that there was insufficient good quality evidence to demonstrate significant benefits of shared care arrangements [4]. The Cochrane review also indicated that further work in this area would benefit from examining which ‘components’ of shared care are effective in order to determine the settings and patient groups in which shared care may be most effective. The review only reviewed economic studies if they were related to the included effectiveness studies, considerably limiting the scope of cost-effectiveness evidence reviewed.

### Shared care remains a current and important idea in care delivery

Although the Cochrane review authors suggested that there is ‘no evidence to support the widespread introduction of shared care services at present’ [4], shared care or similar integrated/collaborative care arrangements continue to be used. More generally, initiatives like the electronic patient record and the national Connecting for Health programme suggest that it is still important to have effective ways to share care information between different organisations and sectors of health-care provision. Investigating who shared care works for, in what circumstances and why will potentially support the following current NHS and social care policy goals:

a) Delivery of care closer to home.

b) Quality, Innovation, Productivity and Prevention aspirations, which intend to achieve efficiencies through delivering the right health care service, in the right place at the right time. QIPP focuses on better quality health care and increasing the throughput of patients, in ways which are innovative and that look to add value by preventing further (more expensive) health and care service interventions.

c) Personalisation. There is increasing emphasis on the importance of tailoring health and care services to the needs and aspirations of individual patients. Personalisation refers to a range of mechanisms that are intended to put members of the public more firmly in control of what care they receive, how it is delivered, in what settings and what instances. Shared care can be viewed as a way to deliver more personalised care, as it can involve mutual decision-making with patients.

### The need to incorporate and synthesise economic evidence

Systematic reviews that assess the cost-effectiveness of complex interventions are being encouraged to take a more contextualised and explanatory focus. There are two central research needs in relation to the conduct of systematic reviews of economic studies:

1. to ‘look beyond the established methodological conventions of systematic reviewing … [to] focus less on producing a pooled estimate of outcomes and mor*e* on *explaining why certain programmes work* (and conversely, *why*, *sometimes*, *they don*’*t*), or are more or less costly or cost-effective; and to

2. develop review methods that recognise that health and other outcomes are jointly produced by both the interventions themselves and the multi-level contexts in which they are placed’ ([8,9])

Therefore a secondary, more methodological aim of this study is to extend and adapt the approach of realist reviews to provide evidence of cost-effectiveness and resource use, as well as effectiveness. We think this will be particularly useful in relation to shared care because some of the underlying programme theories may be inherently economic; they may be as much about expected resource use or cost savings as they are about intended improvements in care outcomes.

This systematic review protocol is registered on the PROSPERO database (registration number: CRD42012002842).

### Review aim

This study aims to explain the effectiveness and cost-effectiveness of shared care using realist review methodology. The review will have two phases.

Phase 1 will identify programme theories about how shared care works, for whom it works, in what contexts and why (ideas about what enables or inhibits effective care sharing between different services and/or professionals) from a range of published and other sources.

Phase 2 will test these programme theories, using published and unpublished empirical evidence, through the process of reasoning detailed under ‘Data synthesis’.

### Review objectives

1. Examine the evolution of the concept of shared care

2. Develop a range of programme theories that describe how shared care works, who it works for, in what circumstances and why.

3. Identify and describe the most important mechanisms by which shared care is thought to produce better outcomes for patients, carers or health and other care services

4. Identify and describe the contextual factors which augment/activate or decrease/negate the impact of these mechanisms (by which shared care is thought to produce better outcomes for patients, carers or health and other care services)

5. Identify and describe the resource use and cost requirements or impacts of the different mechanisms and contextual factors

6. Investigate the effectiveness and cost-effectiveness of shared care using realist review methodology in order to explain better the circumstances in which shared care is likely to be effective and cost-effective.

## Methods

Realist reviews are systematic reviews, in the sense that they have explicit research questions and methods for identifying, selecting, appraising relevant studies, papers or reports and also methods of evidence synthesis. Accordingly realist reviews exhibit the standard phases of systematic review, and these are described below. A key output from a realist synthesis is a programme theory or theories of the intervention in question. This is achieved through specifying how and why the programme or service is thought to cause its intended outcomes (Phase 1: theory building) through close examination of the active mechanisms and contexts, and then testing the assumptions of that theory against further evidence, in order to strengthen and refine it (Phase 2: theory testing). The process at this stage seeks out and incorporates or eliminates rival explanations for the observed outcome pattern. In contrast to conventional systematic reviews of effectiveness studies, there is likely to be greater overlap of these phases, as well as some iteration, as new evidence is sourced that has implications for earlier phases of the review process.

Programme theories are possible explanations for the way in which particular interventions are thought to work; they describe the way in which change occurs because of an intervention. Realist reviews are concerned with unearthing the underlying mechanisms of action that make interventions work, and through looking at the contexts they are in, and the outcomes generated, the reviewer theorises on what is happening in the intervention and why. Mechanisms can be defined for how a programme’s resources or opportunities interact with the reasoning of individuals and lead to changes in behaviour and other causal factors. Contexts are the wider configuration of factors, not necessarily connected to a programme, which may enable or constrain the operation of specific mechanisms.

Another important feature of our review method will be the involvement of a Project Reference Group (PRG) made up of patients with experience of shared care and their carers, and a selection of people who manage or provide shared care arrangements. The group serves two purposes. Firstly, it aids the process of theory development and refinement, and secondly it ensures that the research findings are clear and useful to those involved in managing or providing services for people with chronic diseases.

### Phase 1: theory initiation and development

Phase 1 aims to build a programme theory or theories of shared care, which explain how and why shared care is thought to lead to better outcomes for patients or others (for example, service managers or the health system overall).

For developing the programme theory we intend to undertake an initial immersion in sources of information about the effectiveness and cost-effectiveness of shared care in the following ways.

Through ongoing conversations with experts in the field, a mind-map is being developed of ideas, views and opinions on why shared care works, who it works for, in what circumstances and why. This will be used to guide the literature searches.

The references and citations in the 2007 Cochrane Review, the 1994 Hickman taxonomy paper and the 1994 Report for the Royal College of General Practitioners on shared care in diabetes will be used for a literature search.

A search will be made of past and current government policy on shared care. The grey literature, including websites and blogs, will also be searched.

The following types of key word will be used to guide the initial searches (likely to evolve as the search develops). Population-specific key words include ‘long-term conditions’ and ‘diabetes’. Intervention-specific key words are ‘shared care’, ‘integrated care’ and ‘collaborative care’. Intervention-component-specific key words are ‘shared care records’, ‘e-health records’ and ‘referral management’.

The exclusion/inclusion criteria are necessarily broad in order to ensure the theory development phase is able to take in the widest range of evidence of theories. We will exclude studies on children and young people (up to 18). We will also exclude studies with insufficient detail on why the intervention works, for whom, in what respects and why are excluded.

We will include studies on adults and older adults (18+) who have a long-term condition (chronic disease). We will include studies on interventions that involve professionals having shared responsibility or care planning for a defined group of patients (for example with a specific chronic disease). We will also include studies that are likely to help us answer the following question:

What are the theories or mechanisms by which shared care is thought to produce better outcomes for patients, carers or health and other care services?

Where helpful, this may include consideration of the following more specific questions in order to adequately map the complexity of the intervention, its mechanisms and contexts.

1. How is the programme supposed to work?

2. Are there differences in the understanding of what the programme is trying to do?

3. Is the programme theory applied consistently and cumulatively?

4. Does the programme theory tend to bend (evolve) in actual use?

5. Does the programme theory fare better with particular individuals, interpersonal relations, institutions and infrastructures?

6. Does the policy apparatus surrounding the theory advance or impede it?

7. Is the theory self-affirming, self-defeating or self-neutralising (for example over time)? [6]

In addition to the above questions, because this review is looking at cost-effectiveness, we will also be looking at the evidence from the perspective of the following question,

What are the resource use and cost requirements or impacts of the different mechanisms and contextual factors (that are thought to explain the effectiveness of shared care)?

### Data extraction (Phase 1)

For Phase 1 of the review, sources that contribute to theory development will be reviewed by the lead reviewer through a process of note-taking, annotation and conceptualisation. At the same time we will refine our conceptualisation of shared care through building a programme theory of shared care. Note-taking will be structured and tabulated in a way that best enables dialogue amongst the review team and transparency about which sources informed which strands of theoretical development.

### Quality assessment (Phase 1)

A formal quality assessment will not be undertaken as part of Phase 1. Instead, the lead reviewer will make judgments about the relevance of each source in contributing to theory development on a source-by-source basis, providing explanation of why and to what extent the source is thought to be relevant.

### Formalising the programme theories (data synthesis)

The final part of the first phase is to use our accumulated knowledge from the obtained sources to create either some form of explanatory model and/or a short-list of candidate theoretical mechanisms and related contexts. The origins of these ideas, and the specific sources on which they are grounded, will be documented and justified. From this explanatory model, further questions will be developed that will be used to guide the next phase of the review and search. At this phase, the model needs a degree of middle-range abstraction and conceptualisation to ensure that it is identifiable in sources and therefore testable – there is no point specifying too closely at this phase as that would likely close off potentially useful avenues of sources.

### Phase 2: theory refinement/testing

#### Search strategy

The search strategy is as for Phase 1, but in particular it will seek evidence from empirical studies (qualitative or quantitative) of deviant or rival explanations to the theory developed. In addition, we will identify and include quantitative comparative studies in order to examine any associations between effectiveness/lack of effectiveness and the presence and strength of presence of the theories/mechanisms. For this part of the evidence synthesis we will include: randomised controlled trials, non-randomised controlled trials, controlled before-and-after studies and uncontrolled before-and-after studies. The theory testing/refinement phase of the review may also make use of primary qualitative research studies or mixed-methods research where relevant. The point throughout is to ensure that the programme theory that is being tested determines what evidence is sought (that is, it is based on relevance), in order to provide data that enables theory development and testing.

### Data extraction (Phase 2)

For phase 2, we expect a set of basic information will be collected from relevant groups of included studies. These tables and data fields will be developed from Phase 1, based on the formalised programme theories. Other less standard data of relevance to the specified programme theories will also be extracted. This data will be extracted by one reviewer and a sample of forms checked by the second reviewer. Disagreements or discrepancies will be resolved through involvement of the third member of the review team.

The data-extraction process itself will involve critical discussion between reviewers and the wider team so that data are not simply classified but are used to begin to develop a line of argument that feeds into the final synthesis phase.

### Quality assessment (Phase 2)

Realist reviews are mixed-methods reviews as they incorporate both quantitative and qualitative studies, as well as grey literature. Therefore, different approaches to quality assessment are necessary during the review. Overall, the quality of evidence will be judged by how well it meets the explanatory need of the review at that time: it will be judged on how relevant it is to theory building and refining (during Phase 1) and how far it added rigour to (tested) the theory (during Phase 2).

For particular study types, we intend to use a range of quality assessment or critical appraisal tools as appropriate to the study design. For example, when we are assessing the internal validity of comparative effectiveness studies, we intend to use a standard tool for assessing risk of bias and for other study types, such as process evaluations, we intend to assess them using a modified version of the Wallace *et al*. quality appraisal tool for qualitative studies [10]. For the purposes of transparency, we shall document our decisions about rigour (as appraised by the tools) and relevance (the contribution the piece makes to the review as a whole).

### Data synthesis (Phase 2)

For synthesis, a similar strategy will be used for both Phase 1 (identification of programme theories) and Phase 2 (testing of programme theories).

Synthesis of the diverse sources of evidence included in a realist review is conducted through a process of reasoning that is structured around the following activities [6].

a) Juxtaposition of sources of evidence - for example, where evidence about implementation in one source enables insights into evidence about outcomes in another source.

b) Reconciling of sources of evidence - where results differ in apparently similar circumstances, further investigation is appropriate in order to find explanations for why these different results occurred.

c) Adjudication of sources of evidence - on the basis of methodological strengths or weaknesses.

d) Consolidation of sources of evidence - where evidence about mechanisms and outcomes is complementary and enables a multi-faceted explanation to be built.

e) Situating sources of evidence - where outcomes differ in particular contexts, an explanation can be constructed of how and why these outcomes occur differently.

The transparency of a synthesis in a realist review is achieved by documenting these reasoning processes, describing how they are grounded in the empirical evidence and justifying the inferential shifts that occur through this engagement with the evidence.

In addition, for Phase 2, we aim to code studies according to both the presence or absence of selected theoretical mechanisms and contexts, and the broad strength of the findings in relation to shared care outcomes (for example as highly effective, effective or not effective). These combinations of mechanisms, contexts and measures of effectiveness may be used to try to explain between-study (that is between-programme) differences in programme effectiveness. We hope that this might be achieved through tabulating these data and findings, graphically exploring potential associations or possibly more formal techniques such as qualitative comparative analysis.

### Comparator/control

For the first phase of the review, which primarily concerns development of programme theory, there is no need to define comparators or control groups. For the phase of the review that aims to test/refine the programme theories in relation to evidence about patient outcomes from effectiveness studies, the comparators may be any pre-existing or co-existing forms of care in which the care was not formally shared between the relevant professionals or care settings (for example the same types of patient might have been cared for predominantly via hospital specialist outpatient appointments, or in general practice but without planned specialist input).

### Project reference group

As well as being based on documentary sources and their synthesis, as described in this protocol, the selection and prioritisation of programme theories will be informed by two or three meetings with a Project Reference Group. The PRG will be made up of people who have long-term conditions, and their carers, who have experienced shared care, as well as professionals who have experience of sharing care. The PRG will help identify and choose between different potential programme theories, and also provide a reality check on the clarity and explanatory potential of the selected theories/mechanisms, which will become a focus of Phase 2 of the review.

### Dissemination

The main results of the review will be published and disseminated through the National Institute for Health Research (NIHR) Collaboration for Leadership in Applied Health Research & Care (CLAHRC) for the South West Peninsula. The review will be distributed to relevant teams at the Department of Health (for example the Long Term Conditions Quality, Innovation, Productivity and Prevention Team), as well as to Clinical Commissioning Groups within the geographical area of the South West Peninsula. The review will also be distributed to NHS Commissioning and Providing organisations, as well as appropriate non-statutory health or care service providers. The work will also be presented at a relevant national conference, and publications from the review will be written up and published in peer-reviewed academic journals.

## Discussion

This realist review will explore why and for whom shared care works, in order to support decision-makers working to improve the effectiveness of care for people outside hospital. The development of realist review methods to take into account cost and cost-effectiveness evidence is particularly innovative and challenging, and if successful will offer a new approach to synthesising economic evidence.

## Abbreviations

CLAHRC: Collaboration for Leadership in Applied Health Research and Care;NHS: National Health Service;NIHR: National Institute for Health Research;PRG: Project reference group;QIPP: Quality, innovation, productivity and prevention

## Competing interests

The authors declare that they have no competing interests.

## Authors’ contributions

RH led the design and drafting of the review protocol. MP contributed to the design and drafting of the review protocol. RH scoped and designed the search strategy in collaboration with Chris Cooper, Information Specialist. RB provided substantive topic-specific input that informed the revision and refinement of the review protocol. RA conceived the review and oversaw the development and revision of the protocol, and MP provided substantive methodological input. All authors read and approved the final manuscript.
